# Aspectos genéticos e imagenológicos de la enfermedad quística renal en pediatría: serie de casos

**DOI:** 10.7705/biomedica.7110

**Published:** 2024-05-31

**Authors:** Rafael Adrián Pacheco-Orozco, Jessica María Forero-Delgadillo, Vanessa Ochoa, Juan Sebastián Toro, Harry Pachajoa, Jaime Manuel Restrepo

**Affiliations:** 1 Servicio de Genética, Fundación Valle del Lili, Cali, Colombia Fundación Valle del Lili Fundación Valle del Lili Cali Colombia; 2 Servicio de Nefrología Pediátrica, Fundación Valle del Lili, Cali, Colombia Fundación Valle del Lili Fundación Valle del Lili Cali Colombia; 3 Servicio de Imágenes Diagnósticas, Fundación Valle del Lili, Cali, Colombia Fundación Valle del Lili Fundación Valle del Lili Cali Colombia; 4 Facultad de Ciencias de la Salud, Universidad Icesi, Cali, Colombia Universidad Icesi Universidad Icesi Cali Colombia; 5 Centro de Investigaciones en Anomalías Congénitas y Enfermedades Raras (CIACER), Universidad Icesi, Cali, Colombia Universidad Icesi Universidad Icesi Cali Colombia

**Keywords:** enfermedades renales poliquísticas, riñón poliquístico autosómico recesivo, riñón poliquístico autosómico dominante, pediatría, genética, radiología, diagnóstico por imagen, Polycystic kidney diseases, polycystic kidney, autosomal recessive, polycystic kidney, autosomal dominant, pediatrics, genetics, radiology, diagnostic imaging

## Abstract

Las enfermedades quísticas renales son condiciones frecuentes cuya etiología puede ser muy heterogénea, por lo que se requiere un adecuado abordaje para su diagnóstico y manejo. El objetivo de este trabajo fue ilustrar parte del espectro de la enfermedad renal quística por medio de casos clínicos manejados en la Fundación Valle del Lili. Se describen 11 casos clínicos que incluyen enfermedades como displasia multiquística renal, enfermedad poliquística renal autosómica dominante y autosómica recesiva, entre otras. Las enfermedades quísticas renales varían en su presentación clínica, historia natural, hallazgos imagenológicos, bases genéticas y fisiopatológicas, por consiguiente, el enfoque diagnóstico y el manejo integral se debe realizar de forma individualizada y con un abordaje multidisciplinario.

## Abstract (en)

Renal cystic diseases are common conditions whose etiology can be highly heterogeneous. They require a correct approach for adequate diagnosis and management. We aimed to illustrate part of the spectrum of renal cystic diseases through some clinical cases managed in our service. We describe 11 clinical cases including clinical entities such as renal multicystic dysplasia, and autosomal dominant and autosomal recessive polycystic renal disease, among other pathologies. Renal cystic diseases are heterogeneous in their clinical presentation, natural history, radiological findings, and genetic and pathophysiological basis. An integral clinical approach is needed to get a clear etiological diagnosis and offer adequate individualized care and follow-up for patients.

Los quistes renales son las lesiones más comunes de las que ocupan espacio, con una frecuencia poblacional que aumenta con la edad y que se estima en el 7 al 10 %, aproximadamente ^1^; corresponden a lesiones cerradas con un recubrimiento epitelial, usualmente llenas de líquido, y que se pueden clasificar según sus características morfológicas, imagenológicas o etiológicas ^2^.

Las lesiones quísticas se dividen en simples y complejas. Las simples son lesiones discretas de forma ovalada o circular que tienen una pared lisa, delgada y bien definida, sin componente sólido, tabiques o calcio en su interior. Su apariencia ecográfica es anecoica y, en tomografía axial, es homogéneamente hipodensa. En la resonancia (RM), los quistes simples se caracterizan por ser hipointensos en T_1_ e hiperintensos en T_2_, sin realce interno tras el contraste, llenos de un trasudado homogéneo de baja densidad. Estos quistes tienden a aumentar en tamaño y número con la edad y, usualmente, se encuentran de manera incidental en imágenes abdominales ^1^. Cuando una lesión quística renal no cumple con los criterios necesarios para ser clasificada como un quiste simple, se le denomina compleja o atípica ^3^.

En la clasificación de Potter, se proponen cuatro tipos de enfermedades renales quísticas: el tipo 1 o enfermedad renal poliquística infantil (enfermedad poliquística renal autosómica recesiva), el tipo 2 o enfermedad renal displásica quística (enfermedad renal displástica multiquística), el tipo 3 o enfermedad poliquística renal del adulto (enfermedad poliquística renal autosómica dominante), y el tipo 4, por obstrucción parcial o intermitente del flujo urinario (displasia obstructiva) ^4^. En la clasificación de Liapis y Winyard, se reconocen ocho grupos de enfermedades renales quísticas: enfermedad renal poliquística, quistes medulares renales, quistes en síndromes de cáncer hereditario, quiste renal multilocular, enfermedad quística localizada, quistes corticales simples, quistes adquiridos y los misceláneos ^5^.

La etiología de las enfermedades renales quísticas incluye un amplio espectro de condiciones hereditarias, congénitas o adquiridas, y requiere un abordaje multidisciplinario para su adecuado diagnóstico y manejo ^2^. El desarrollo renal es un proceso muy bien regulado que implica interacciones complejas entre tejidos, y se puede dividir en procesos que ocurren durante la nefrogénesis y después de ella ^6^. El proceso de nefrogénesis se puede dividir en inducción de la yema ureteral, ramificación del uréter, transición epitelio-mesénquima, e inducción y formación de patrones de la nefrona ^6^^,^^7^. La formación de quistes o cistogénesis ocurre después de la nefrogénesis, por defectos en la preservación de la estructural renal ante el estrés mecánico y el funcional ^7^. Esta cistogénesis está relacionada con alteraciones en las proteínas involucradas en la traducción de señales asociadas con la proliferación y la muerte celular, que usualmente están localizadas en el cilio apical primario, las uniones intercelulares y las adhesiones focales ^8^.

El objetivo de este artículo fue ilustrar parte del amplio espectro de la enfermedad renal quística, con una serie de casos clínicos provenientes de Colombia que representan situaciones de la práctica médica.

## Presentación de casos

Se seleccionaron 11 casos clínicos de pacientes que presentaban compromiso por enfermedad quística renal. Los casos se seleccionaron a discreción de los autores con base en los pacientes atendidos en el Servicio de Nefrología Pediátrica de la Fundación Valle del Lili. Se recopiló la información clínica y las imágenes de cada caso a través del sistema electrónico de historia clínica de la institución (cuadro 1).

**Cuadro 1:** Resumen de los casos clínicos

Caso clínico	Presentación clínica	Hallazgos radiológicos	Hallazgos genéticos
Displasias			
Displasia renal multiquística	Falla renal aguda en el contexto de una infección gastrointestinal a los dos años	- Riñón izquierdo de apariencia displásica multiquística	No solicitado
		- Riñón único derecho hidronefrótico por obstrucción secundaria a una estenosis de la unión pieloureteral	
Nefropatía por PAX2	- Oligohidramnios	Aumento de la ecogenicidad renal con quistes corticales periféricos milimétricos	*PAX2* c.94C>T p.Pro32Ser
	- Agenesia renal derecha		Heterocigosis
	- Displasia renal izquierda		
Nefropatía por HNF1B	Diabetes de tipo 1 a los 11 años de edad	- Riñones poliquísticos	Deleción heterocigota en la región 17q12 incluyendo el gen *HNF1B*
		- Útero bicorne	
Enfermedad fibroquística hepatorrenal			
Enfermedad poliquística renal autosómica recesiva	- Criptorquidia bilateral	Múltiples imágenes quísticas bilaterales con ecogenicidad heterogénea y pérdida de la diferenciación corticomedular	-*PKHD1* c.1342G>C p.Gly448Arg Heterocigosis
	- Diástasis de rectos abdominales		-*PKD1* c.7906C>T p.Arg2636Trp
	- Pielonefritis a los seis meses de vida		Heterocigosis
Enfermedad poliquística renal autosómica recesiva	- Inicio de terapia de reemplazo renal a los seis años	Nefromegalia bilateral asociada con poliquistosis renal bilateral	Panel NGS negativo
	- Nefrectomía bilateral y trasplante renal a los 8 años		
Enfermedad poliquística renal autosómica dominante de inicio muy temprano	Sin compromiso clínico	- Múltiples imágenes quísticas desde la semana 23 de gestación	Panel NGS negativo
		- Al nacimiento, más de 10 quistes parenquimatosos bilaterales con aumento de la ecogenicidad, pérdida de la diferenciación corticomedular y disminución del tamaño del riñón izquierdo	
Enfermedad poliquística renal autosómica dominante	Padre con enfermedad poliquística renal y enfermedad renal crónica en estadio IV	Quistes simples corticales bilaterales que aumentan con el tiempo	Pendiente de valoración genética
Nefronoptisis de tipo 3	- Antecedente familiar de hermano fallecido por síndrome hepatorrenal	Alteración de la ecogenicidad renal y signos de nefropatía crónica	*NPHP3* c.2571_2574del p.Ser857Argfster4 Homocigosis
	- Elevación de transaminasas a los nueve meses		
	- Falla renal en estadio V a los dos años		
	- Trasplante hepatorrenal a los tres años		
Síndrome de Joubert	Enuresis diurna con poliuria y polidipsia	Nefromegalia con pérdida de la diferenciación corticomedular	*RPGRIP1L*
			- c.697A>T p.Lys233Ter
			- c.3545delC p.Pro1182LeufsTer25
Otros			
Neonato pretérmino	- Nacimiento a las 26 semanas de gestación	Riñones con aumento de ecogenicidad cortical, múltiples imágenes quísticas corticales bilaterales, menores de 5 mm	No solicitado
	- Deficiencia de vitamina D, múltiples fracturas e hipocalcemia grave		
Hidronefrosis obstructiva	- Nacimiento a término sin complicaciones	- Ecografía prenatal con hidronefrosis bilateral	No solicitado
	- Infección de vías urinarias por Citrobacter freundii a los tres meses	- Dilatación grave de los sistemas colectores, abombamiento de los cálices, adelgazamiento del parénquima renal y dilatación de los uréteres	

Para la descripción de los casos se plantea una organización que corresponde a tres grandes grupos de enfermedad quística renal, así: displasias, enfermedad fibroquística hepatorrenal y otros ^5^.

## Displasias

### Displasia multiquística

**Figura 1:**
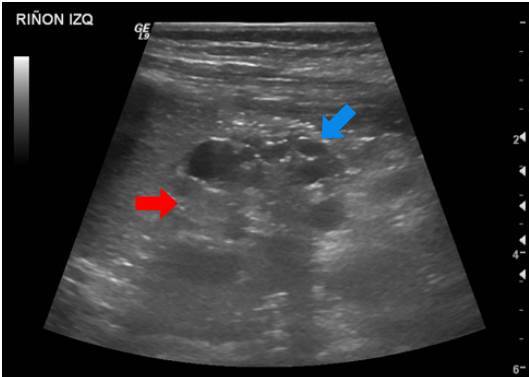
Displasia renal multiquística. Además de los quistes renales simples (flecha azul), de localización cortical, se observa pérdida de la diferenciación corticomedular y aspecto hipotrófico del riñón izquierdo (flecha roja).

Se trató de un paciente de sexo masculino que a los dos años de vida presentó un cuadro clínico de lesión renal aguda KDIGO 3 en el contexto de una infección gastrointestinal ^9^. Se practicó una ecografía renal que evidenció la dilatación de las vías urinarias del lado derecho, por obstrucción secundaria a estenosis de la unión pieloureteral, con un riñón izquierdo de apariencia displásica multiquística (figura 1).

Además, el Servicio de Cirugía Pediátrica le practicó una pielostomía derecha. En el procedimiento quirúrgico, se evidenció una dilatación de la pelvis renal de 8 cm de diámetro, aproximadamente, con estenosis completa de la unión pieloureteral derecha.

Posterior al procedimiento, el paciente ha tenido seguimiento regular por nefrología pediátrica, con compromiso inicial posoperatorio de enfermedad renal crónica en estadio 3 y con una tasa de filtración estable de 40 ml/ min/1.73 m^2^. Además, durante su evolución, desarrolló hipertensión arterial sistémica y deterioro clínico al estadio 4 a los 10 años de vida. Actualmente, tiene 12 años y se encuentra cumpliendo el protocolo de trasplante renal.

*Análisis*. Los riñones displásicos multiquísticos son masas renales no funcionales, causados por alteraciones en la diferenciación metanéfrica y caracterizados por la presencia de múltiples quistes que reemplazan en su totalidad el parénquima renal. Usualmente, se visualizan en la evaluación ecográfica prenatal; hay afectación de un solo riñón y tienden a involucionar en el útero o después del nacimiento en el 95 % de los casos. Esta condición suele ocurrir de manera esporádica; sin embargo, se han visto casos donde hay agregación familiar o puede presentarse en el contexto de condiciones multisistémicas por mutaciones en los genes *EYA1*, *SIX1*, *HNF1B* y *PAX2*, entre otros ^10^.

### Nefropatía por mutación en PAX2

**Figura 2:**
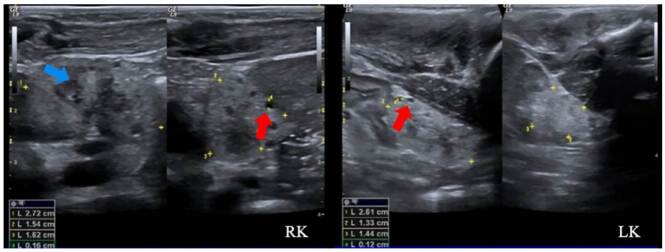
Nefropatía por PAX2 (síndrome coloboma-renal). Hipoplasia y displasia de ambos riñones con pérdida de la diferenciación corticomedular (flecha azul) y aumento de la ecogenicidad cortical, con presencia de múltiples quistes simples de tamaño menor de 10 mm (subcentimetric) (flechas rojas)

Se trató de una paciente de sexo femenino, producto de una unión no consanguínea, sin antecedentes familiares de relevancia, con hallazgos prenatales de oligohidramnios, agenesia renal derecha y displasia renal izquierda. La ecografía renal posnatal evidenció aumento de la ecogenicidad renal con quistes corticales periféricos milimétricos, lo cual sugirió el diagnóstico de enfermedad poliquística renal autosómica recesiva (figura 2). La secuenciación del exoma clínico reveló la presencia de una variante patogénica de *novo* en el gen *PAX2*, previamente reportada ^11^.

*Análisis*. El gen *PAX2* codifica para un factor de transcripción esencial en el desarrollo del epitelio renal ^12^, especialmente para el ensamblaje del conducto mesonéfrico y la yema ureteral ^13^. Las mutaciones de este gen se han asociado principalmente con el síndrome de coloboma renal o papilorrenal (MIM 120330). Sin embargo, se sabe que puede causar un espectro fenotípico amplio que va desde anomalías congénitas del riñón y el aparato urinario (*Congenital Anomalies of the Kidneys and Urinary Tract*, CAKUT) hasta la enfermedad quística renal, junto con una gran variedad de manifestaciones extrarrenales ^11^^,^^14^.

### Nefritis tubulointersticial por mutación en HNF1B

Se trató de una paciente de sexo femenino con diagnóstico de riñones poliquísticos a los 9 años, de diabetes de tipo 1 a los 11 años y de útero bicorne a los 28 años, con el antecedente del mismo fenotipo en su madre, abuela y bisabuela materna. Había sido sometida en dos ocasiones a trasplante renal y en una biopsia se reportó nefritis tubulointersticial. Se le realizó un exoma clínico único que evidenció una deleción heterocigota en la región 17q12 que incluye el gen *HNF1B*, confirmada por *Array Comparative Genomic Hybridization* (CGH).

*Análisis*. La nefritis tubulointersticial corresponde a la presencia de lesiones inflamatorias de los túbulos renales y el intersticio, y se caracteriza histopatológicamente por infiltrado inflamatorio, edema y fibrosis. Se puede presentar de manera aguda o crónica y su etiología incluye causas infecciosas, medicamentosas, inmunológicas, tóxicas y hereditarias ^8^.

El gen *HNF1B* está localizado en el cromosoma 17 y codifica para el factor nuclear hepatocítico 1β, que cumple un papel importante en el desarrollo embrionario de múltiples sistemas, especialmente del riñón, el hígado, el páncreas y el tracto genitourinario ^15^. La nefropatía por variantes en el gen HNF1B puede generar un fenotipo muy variable, desde CAKUT y enfermedad renal tubulointersticial, hasta enfermedad renal quística y diabetes ^16^. En el 40 al 50 % de los casos, la alteración molecular es una deleción completa del gen HNF1B como parte del síndrome recurrente de deleción 17q12 ^17^.

## Enfermedad fibroquística hepatorrenal

### Enfermedad poliquística renal de herencia autosómica recesiva

**Figura 3:**
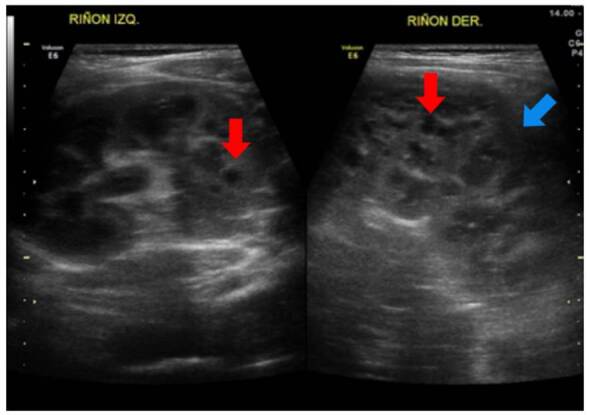
Enfermedad poliquística renal autosómica recesiva. Se observan múltiples imágenes quísticas simples de diferente tamaño, frecuentemente menores de 10 mm (s*ubcentimetric*), localizadas tanto en la médula como en la corteza (flechas rojas). También, hay pérdida de la diferenciación corticomedular (flecha azul). No se observa hipotrofia renal.

Se trató de un paciente de sexo masculino, producto de una unión consanguínea, que a los seis meses de vida presentó un episodio de pielonefritis. La ecografía renal mostró imágenes sugestivas de enfermedad poliquística renal autosómica recesiva, con múltiples imágenes quísticas bilaterales de ecogenicidad heterogénea y pérdida de la diferenciación corticomedular (figura 3). No presentaba lesiones hepáticas en la ecografía y la función hepática era normal.

A los cinco años de vida, una junta médica de genética clínica consideró que se trataba de una secuencia de obstrucción temprana de las vías urinarias (riñón multiquístico, diástasis de rectos abdominales y criptorquidia bilateral), con antecedentes familiares de consanguinidad parental, lo que sugiere una enfermedad poliquística renal de herencia autosómica recesiva. En un panel de secuenciación de nueva generación de los genes *PKD1*, *PKD2* y *PKHD1*, no se encontraron variantes patogénicas en estos, por lo que se solicitó secuenciación de exoma en trío, actualmente en proceso.

Hasta el último control de nefrología, a los 6 años de vida, el paciente se encontraba asintomático, con adecuada función renal, sin proteinuria ni hipertensión arterial.

*Análisis*. La enfermedad poliquística renal de herencia autosómica recesiva es una condición rara y de presentación usualmente grave en los primeros años de vida. Su incidencia se ha reportado como un caso por cada 26.500 recién nacidos vivos ^18^. Representa un espectro clínico que incluye la presencia de quistes que afectan los conductos colectores, así como nefromegalia, pérdida de la diferenciación corticomedular y fibrosis hepática; es causada principalmente por variantes bialélicas del gen de la fibrocistina (*PKHD1*), aunque también se han reportado variantes del gen *DZIP1L* (*DAZ interacting protein 1-like*) ^19^. Sin embargo, existe un gran número de síndromes con presentaciones similares (fenocopias), por lo cual se indica el diagnóstico molecular mediante secuenciación masiva paralela de múltiples genes. Se estima que en el 20 al 25 % de los pacientes no se encuentra la causa genética ^20^.

### Otro caso

**Figura 4:**
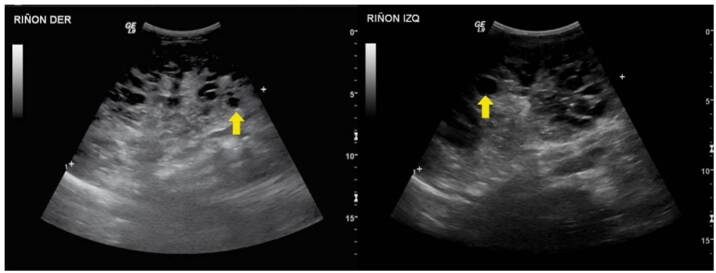
Enfermedad poliquística renal autosómica recesiva. Se observa gran cantidad de quistes simples, de tamaño menor de 10 mm (*subcentimetric*), de distribución aleatoria, que reemplazan casi la totalidad de los riñones (flechas amarillas).

Se trató de una paciente de sexo femenino, producto de una unión no consanguínea, sin antecedentes familiares de enfermedad renal, en quien se documentó nefromegalia bilateral asociada con poliquistosis renal bilateral desde el primer año de vida (figura 4). Se inició terapia de reemplazo renal a los seis años de vida, y fue sometida a nefrectomía bilateral y trasplante renal con donante vivo relacionado, a los ocho años. En el estudio histopatológico se informó adelgazamiento cortical con disminución del número de glomérulos y reemplazo del parénquima por lesiones quísticas. Al aplicar un panel de secuenciación de nueva generación para enfermedad poliquística renal, no se demostraron variantes patogénicas asociadas con el cuadro clínico de la paciente. Actualmente, a los 17 años, la paciente continúa en seguimiento con tratamiento inmunosupresor, función renal estable, sin hipertensión arterial y sin alteraciones del estado ácido-base ni de los hidroelectrolíticos. Una ecografía abdominal reciente no mostró lesiones hepáticas.

*Análisis*. De los pacientes con enfermedad poliquística renal de herencia autosómica recesiva, entre el 30 y el 40 % fallece por complicaciones asociadas con hipoplasia pulmonar en el periodo neonatal ^2^. El 75 % de los pacientes presenta hipertensión arterial sistémica, predominante en los primeros meses de vida, y el 60 % desarrolla enfermedad renal terminal a los 20 años ^18^.

### Enfermedad poliquística renal autosómica dominante de inicio muy temprano

**Figura 5:**
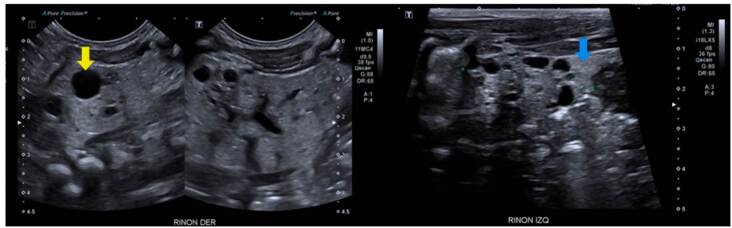
Enfermedad poliquística renal autosómica dominante de inicio muy temprano. Se observan múltiples quistes simples, distribuidos de manera aleatoria, de diferente tamaño, pero más grandes (flecha amarilla) que los de la enfermedad poliquística renal recesiva. También, existe aumento de la ecogenicidad cortical (flecha azul).

Se trata de una paciente de sexo femenino, producto de una unión no consanguínea, sin antecedentes familiares de relevancia. En una ecografía prenatal en la semana 23 de gestación, se observó el riñón izquierdo con múltiples quistes. Al nacimiento, una ecografía renal y de vías urinarias mostró más de 10 quistes parenquimatosos, bilaterales, distribuidos aleatoriamente, y aumento de la ecogenicidad parenquimatosa, pérdida de la diferenciación corticomedular y disminución en el tamaño del riñón izquierdo (figura 5). La ecografía abdominal total no demostró quistes hepáticos. En el estudio de orina se encontró proteinuria leve.

Por las características clínicas, se sospechó una enfermedad poliquística renal autosómica dominante de presentación neonatal. Se realizó secuenciación de exoma dirigida a genes relacionados con poliquistosis renal, pero no se reportaron variantes patogénicas, por lo que se indicó secuenciación de exoma clínico dado el compromiso renal asociado con manifestaciones extrarrenales, como su fenotipo facial y convulsiones.

*Análisis*. La enfermedad poliquística renal autosómica dominante es la enfermedad quística renal hereditaria más común, con una incidencia aproximada de dos casos por 1.000 recién nacidos vivos y es una de las principales causas de enfermedad renal terminal ^2^. Se caracteriza por el desarrollo y expansión progresiva de múltiples quistes a lo largo del parénquima renal, con deterioro paulatino de la función renal que conlleva enfermedad renal terminal cerca de la sexta década de la vida. Usualmente, se diagnostica hacia la segunda o tercera décadas de la vida. Sin embargo, existen presentaciones más tempranas, como la enfermedad poliquística renal autosómica dominante de inicio temprano, que se observa entre los 18 meses y los 15 años; y la enfermedad poliquística renal autosómica dominante de inicio muy temprano, con hallazgo de quistes antes de los 18 meses de vida. Este último grupo corresponde a menos del 1 % de los casos de este tipo de enfermedad poliquística ^22^.

### Enfermedad poliquística renal autosómica dominante

**Figura 6:**
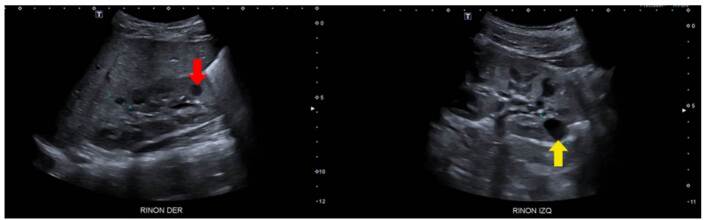
Enfermedad poliquística renal autosómica dominante. Se observan quistes simples de mayor tamaño en médula (flecha roja) y corteza (flecha amarilla), asociados con aumento de la ecogenicidad cortical.

Se trató de un paciente de sexo masculino, asintomático, con antecedente de padre con enfermedad poliquística renal y enfermedad renal crónica en estadio IV ^23^. El seguimiento se inició a los cuatro años de vida con pruebas de función y ecografías renales periódicas. Las ecografías mostraron inicialmente un quiste cortical simple con subsecuente aparición, en nuevas imágenes, de masas quísticas bilaterales con aumento de tamaño (figura 6). Tiene una hermana en seguimiento por el mismo antecedente, también con progresión de lesiones quísticas sin deterioro de la función renal. El paciente continuó con seguimiento institucional hasta el año 2021 (ocho años de vida), momento en el que se encontraba en trámite la autorización por parte de la entidad para la valoración genética.

*Análisis*. La enfermedad poliquística renal autosómica dominante es causada principalmente por variantes patogénicas de los genes que codifican para policistinas, PKD1 (80 % de los casos) o PKD2 (15 %) ^22^. Estos genes codifican para receptores que interactúan entre sí y que son importantes en la regulación de las concentraciones de calcio y la traducción de señales extracelulares en el cilio primario ^24^. Además de sus manifestaciones renales, la enfermedad poliquística renal autosómica dominante se caracteriza por un espectro de manifestaciones extrarrenales -incluyendo enfermedad poliquística hepática y aneurismas intracraneales, entre otras-, por lo que es una enfermedad de compromiso sistémico ^22^^,^^25^. El diagnóstico etiológico de la esta enfermedad es importante porque permite un adecuado seguimiento y la búsqueda de otras manifestaciones de la enfermedad, así como la identificación y tamizaje de familiares en riesgo ^26^.

### Nefronoptisis

**Figura 7:**
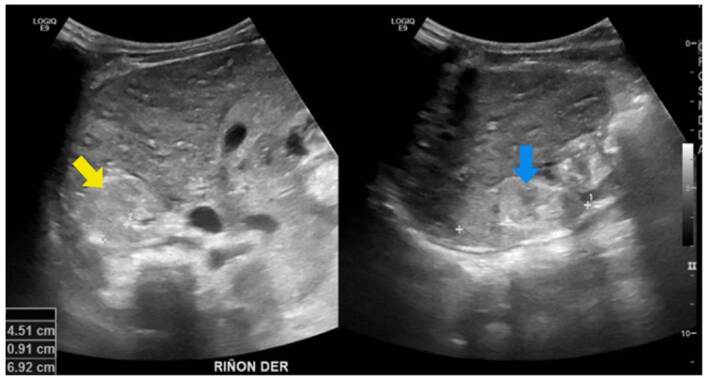
Nefronoptisis de tipo III. En este caso, ya existen cambios de nefropatía crónica, como disminución del tamaño renal, con pérdida importante de la diferenciación corticomedular, aumento de la ecogenicidad (flecha amarilla) y disminución del espesor cortical (flecha azul).

Se trató de un paciente de sexo masculino, con antecedentes de un hermano que falleció por síndrome hepatorrenal y cuya valoración se inició a los nueve meses de edad por elevación de las transaminasas y ecografía abdominal que evidenció cambios sugestivos de hepatopatía crónica. Posteriormente, se identificó compromiso renal mediante imágenes que mostraron alteración de la ecogenicidad renal y signos de nefropatía crónica (figura 7). A los dos años, progresó rápidamente a enfermedad renal crónica en estadio V y fue sometido a trasplante hepatorrenal a los tres años. Se realizó secuenciación de exoma clínico que detectó la variante patogénica c.2571_2574del p.Ser857Argfster4, homocigota en el gen NPHP3, lo que confirmó el diagnóstico de nefronoptisis de tipo III, de herencia autosómica recesiva. En su último control, a los cuatro años de vida, tenía un adecuado crecimiento y buena función del injerto renal, sin hipertensión arterial.

*Análisis*. La nefronoptisis es una enfermedad renal tubulointersticial de herencia autosómica recesiva ^27^. Se caracteriza por la presencia de nefritis tubulointersticial crónica, de quistes en la unión corticomedular y progresión a falla renal antes de los 30 años, de hecho, es la primera causa de enfermedad renal terminal en las primeras décadas de la vida ^28^^-^^30^. Además, del 10 al 20 % de los casos pueden estar acompañados de manifestaciones extrarrenales o presentarse en el contexto de otras ciliopatías. Según la edad de aparición de enfermedad renal terminal, se clasifica en infantil, adolescente o juvenil ^28^^,^^31^. Se han descrito más de 25 genes relacionados con nefronoptisis; el más frecuente es NPHP1 (nefrocistina 1), presente en 20 a 25 % de los casos. En 60 a 70 % de los casos, no se encuentra una causa genética ^28^.

### Síndrome de Joubert

**Figura 8:**
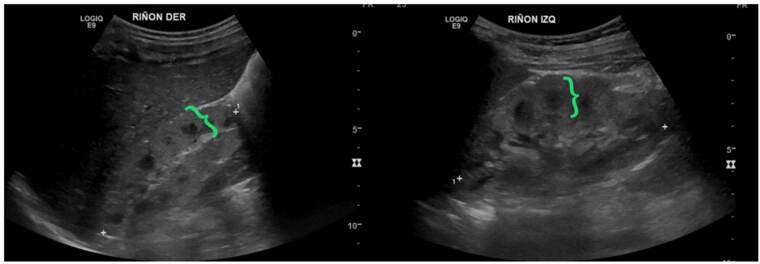
Síndrome de Joubert. Se denota aumento del espesor parenquimatoso (llaves verdes) y longitudinal de ambos riñones para la edad del paciente.

Se trató de un paciente de sexo masculino, producto de una unión no consanguínea, valorado por nefrología pediátrica por enuresis diurna, poliuria y polidipsia, asociadas con aumento del tamaño de los riñones y pérdida de la diferenciación corticomedular (figura 8). El paciente cursó con déficit cognitivo, síndrome dismórfico y polidactilia preaxial en la mano izquierda. El estudio genético evidenció las variantes patogénicas c.697A>T p.Lys233Ter y c.3545delC p.Pro1182LeufsTer25 en heterocigosis compuesta en el gen RPGRIP1L (*Retinitis Pigmentosa GTPase Regulator Interacting Protein 1-Like*), que confirman el diagnóstico de síndrome de Joubert, de herencia autosómica recesiva. En la última valoración por nefrología, a los 16 años, el paciente se encontraba con enfermedad renal crónica en estadio III e hipertensión arterial sistémica.

*Análisis*. El síndrome de Joubert es una ciliopatía caracterizada por un fenotipo neurocognitivo, con hipotonía y signo del molar en la resonancia magnética cerebral ^27^. Estos pacientes también pueden cursar con enfermedad renal quística, polidactilia, distrofia retiniana, coloboma o fibrosis hepática. Tiene una incidencia aproximada de un caso por cada 80.000 a 100.000 recién nacidos vivos y es de herencia autosómica recesiva. Se han descrito mutaciones en más de 20 genes, asociadas con el síndrome de Joubert, y todas implicadas con proteínas del cuerpo basal y el cilio primario ^32^.

## Otros

### Hiperecogenicidad del prematuro

**Figura 9:**
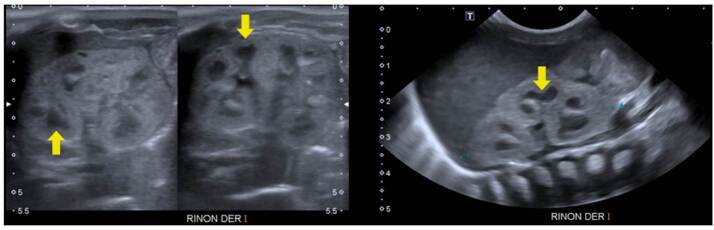
Recién nacido prematuro. Se observa indentación hacia el interior (flechas amarillas) e hipoecogenicidad de las pirámides medulares respecto a la corteza como hallazgo normal esperado para la edad.

Se trató de una paciente de sexo femenino, producto de la primera gestación de la madre de 16 años, que asistió a tres controles prenatales durante todo el embarazo. La paciente nació a las 26 semanas de gestación por ruptura prematura de membranas y sospecha de corioamnionitis. Tuvo múltiples complicaciones asociadas con la prematuridad, como hemorragia intraventricular, conducto arterioso persistente, displasia broncopulmonar, síndrome convulsivo, resección intestinal por enterocolitis necrosante y falla intestinal secundaria; también, presentó deficiencia de vitamina D, múltiples fracturas e hipocalcemia grave. La ecografía renal mostró aumento de la ecogenicidad cortical renal y múltiples quistes corticales, bilaterales y menores de 5 mm (figura 9). Durante el seguimiento, la paciente tenía presión arterial normal y diuresis adecuada, función renal normal, sin acidosis metabólica ni alteraciones electrolíticas.

*Análisis*. La corteza renal exterior -que contiene los glomérulos y los túbulos contorneados- rodea las pirámides medulares, que contienen las asas de Henle y los túbulos colectores. En el paciente prematuro, se puede observar hipoecogenicidad de las pirámides renales, de origen fisiológico y, usualmente, observada en neonatos y en lactantes hasta los seis meses. Lo anterior, debido a que en este periodo los glomérulos ocupan un volumen más grande de la corteza, existe un mayor componente celular del penacho glomerular y algunas asas de Henle se sitúan en la corteza renal ^33^. Esta hipoecogenicidad relativa de las pirámides medulares, comparada con la corteza, puede ser confundida con quistes renales o hidronefrosis en la ecografía.

### Simulación de quistes por dilataciones

**Figura 10:**
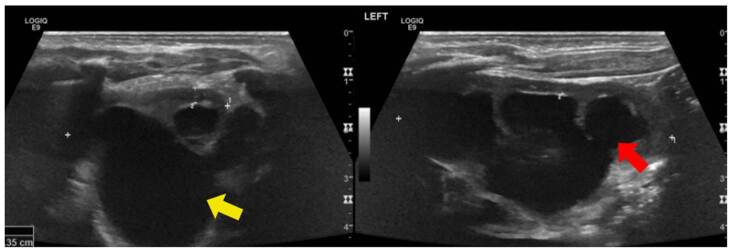
Hidronefrosis obstructiva. A) Dilatación de la pelvis renal (flecha amarilla). B) La comunicación de la pelvis renal con el sistema calicial diferencia la hidronefrosis de lesiones quísticas (flecha roja).

Se trató de un paciente de sexo masculino, con evidencia ecográfica prenatal de dilatación moderada a grave de las vías urinarias. Nació a las 37 semanas de gestación, sin complicaciones. Sin embargo, luego se confirmó la presencia de dilatación grave, compromiso de ambos sistemas colectores y adelgazamiento del parénquima renal y dilatación de los uréteres (figura 10). En la cistouretrografía miccional, se identificaron divertículos vesicales bilaterales en el margen posteroinferior. Se practicó una urorresonancia que mostró estenosis de los uréteres distales, adyacente a las uniones ureterovesicales. A los tres meses de vida, presentó infección de vías urinarias por Citrobacter freundii.

*Análisis*. Los cambios pseudoquísticos renales pueden ser secundarios a dilatación de la pelvis renal y de los cálices por un fenómeno obstructivo. Las principales causas de dilatación de las vías urinarias son el reflujo vesicoureteral y la obstrucción de la unión ureteropélvica ^2^.

La dilatación de la pelvis renal por hidronefrosis obstructiva o reflujo vesicoureteral, puede tener la apariencia de quistes renales.

### Consideraciones éticas

Este estudio fue aprobado por el Comité de Ética en Investigación Biomédica de la Fundación Valle del Lili por medio de la carta de aprobación No. 200- 2023.

## Discusión

Los quistes renales son lesiones con una etiología muy heterogénea que incluye alteraciones hereditarias, del desarrollo y adquiridas del riñón. El diagnóstico diferencial puede ser difícil, dada la variabilidad en la presentación clínica e imagenológica de las lesiones; sin embargo, el establecer el diagnóstico preciso es fundamental para determinar el pronóstico del compromiso renal y obtener información sobre la historia natural de la enfermedad, así como el mecanismo de herencia y la presencia de manifestaciones extrarrenales ^2^.

El primer paso cuando se observan lesiones de apariencia quística en exámenes de imagenología, es establecer si en efecto se trata de quistes o no. Para esto, la ecografía es el método de elección, dado que es costo- eficiente y permite evaluar en detalle la diferenciación corticomedular, las lesiones quísticas, y si existe comunicación entre ellas y la pelvis renal ^34^. Es importante notar que esta metodología depende del evaluador, por lo que debe ser practicada por personal con experiencia en la descripción de hallazgos ecográficos renales. En caso de que se requiera evaluar la anatomía del uréter o descartar la presencia de anomalías congénitas concomitantes del árbol urinario o la vejiga, se puede utilizar una urorresonancia. La tomografía computarizada no tiene indicación en estos pacientes, ya que no brinda un detalle anatómico suficiente y, además, implica una exposición innecesaria a radiación ionizante ^34^.

Una vez confirmado que se trata de quistes, es importante determinar la edad de presentación de las lesiones. Su presentación prenatal, si es unilateral, es sugestiva de riñón multiquístico. En este caso, se espera que el riñón tenga una tendencia a la hipoplasia y disminución progresiva de su tamaño.

Si, por el contrario, el compromiso es bilateral y se ve acompañado de secuencia de oligohidramnios (secuencia de Potter), con nefromegalia e hipoplasia pulmonar, es sugestivo de enfermedad poliquística renal de herencia autosómica recesiva. En estos casos, es importante también descartar una lesión obstructiva en las vías urinarias que configure una hidronefrosis obstructiva.

Además, en raras ocasiones, la enfermedad poliquística renal autosómica dominante puede manifestarse en el periodo neonatal, llamándose en estos casos enfermedad poliquística renal de presentación muy temprana. Una forma de diferenciar la enfermedad poliquística renal autosómica dominante de inicio muy temprano de la de herencia autosómica recesiva, es que en la segunda hay presencia de fibrosis hepática.

Otras enfermedades que se pueden manifestar de manera similar son el grupo de las ciliopatías. Este grupo incluye nefronoptisis, síndrome de Joubert, síndrome de Meckel-Gruber y síndrome de Bardet-Biedl, entre otros ^21^. De estas, la nefronoptisis se caracteriza por quistes en la unión corticomedular y nefritis tubulointersticial crónica, con consecuente deterioro de la función renal. Se puede presentar de manera aislada o en un contexto sindrómico, por lo que, ante hallazgos sugestivos de nefronoptisis, se debe buscar compromiso extrarrenal, como anomalías oculares, alteraciones del sistema nervioso central, polidactilia u obesidad. Además, las variantes patogénicas en genes como *PAX2* o *HNF1B*, así como síndromes de predisposición a tumores (como el complejo de esclerosis tuberosa y el síndrome de von Hippel-Lindau), también se relacionan con diferentes fenotipos de enfermedad quística renal ^21^.

Los antecedentes familiares son de gran ayuda para determinar el probable mecanismo de herencia de la condición. Los antecedentes en familiares directos, como padres, tíos o abuelos, sugieren una herencia autosómica dominante. Este tipo de herencia es vista en la enfermedad poliquística renal autosómica dominante, así como en la nefropatía por mutaciones en *PAX2* o *HNF1B*. En estos casos, el riesgo de recurrencia para la descendencia de un afectado es del 50 %. La herencia autosómica recesiva se sugiere si existe consanguinidad parental, con familiares directos afectados o sin ellos, y es característica de la enfermedad poliquística renal de herencia autosómica recesiva y del grupo de las ciliopatías.

Dado que la presentación clínica e imagenológica de las enfermedades renales quísticas puede ser muy similar, se resalta la importancia de las pruebas genéticas para un adecuado diagnóstico y tratamiento. Este abordaje se puede hacer por secuenciación individual de genes, paneles multigénicos o secuenciación de exoma completo. La selección de la prueba genética dependerá de la sospecha clínica en cada caso. A menos que haya una sospecha clínica muy clara o se conozca la presencia de una variante genética en un familiar, es ideal realizar paneles genéticos que incluyan un gran número de genes asociados con enfermedad quística renal. Se ha reportado un rendimiento diagnóstico del 78 % mediante el uso de paneles genéticos en pacientes con sospecha de enfermedad renal quística hereditaria. En 17 % de los casos, los pacientes no tenían un diagnóstico clínico claro o la prueba genética cambió el diagnóstico ^35^.

En este trabajo se resaltan las diferencias en la presentación clínica, la historia natural de la enfermedad, y las bases genéticas y fisiopatológicas de las diferentes enfermedades renales quísticas, haciendo una correlación con los hallazgos imagenológicos. La amplia heterogenicidad clínica y genética de este grupo de condiciones requiere que el abordaje clínico y paraclínico sea integral, con el objetivo de llegar a un diagnóstico etiológico claro y, así, ofrecer un cuidado y un seguimiento individualizados a los pacientes.
